# Serum CHI3L1 as a biomarker of interstitial lung disease in rheumatoid arthritis

**DOI:** 10.3389/fimmu.2023.1211790

**Published:** 2023-08-17

**Authors:** Rui Yu, Xiaomin Liu, Xiaoyue Deng, Siting Li, Yifei Wang, Yan Zhang, Dan Ke, Rui Yan, Qian Wang, Xinping Tian, Mengtao Li, Xiaofeng Zeng, Chaojun Hu

**Affiliations:** ^1^ Department of Rheumatology, Peking Union Medical College Hospital, Peking Union Medical College and Chinese Academy of Medical Sciences, National Clinical Research Center for Dermatologic and Immunologic Diseases (NCRC-DID), Key Laboratory of Rheumatology & Clinical Immunology, Ministry of Education, Beijing, China; ^2^ Eight-year Medical Doctor Program, Chinese Academy of Medical Sciences & Peking Union Medical College, Beijing, China; ^3^ Department of Rheumatology, Shunyi District Hospital, Beijing, China; ^4^ Medical Science Research Center (MRC), Peking Union Medical College Hospital, Peking Union Medical College and Chinese Academy of Medical Sciences, Beijing, China

**Keywords:** interstitial lung disease, rheumatoid arthritis, chitinase-3 like-protein-1, biomarkers, prediction

## Abstract

**Background:**

Interstitial lung disease (ILD) is a relatively prevalent extra-articular manifestation of rheumatoid arthritis (RA) and contributes to significant morbidity and mortality. This study aimed to analyze the association between chitinase-3 like-protein-1(CHI3L1) and the presence of RA-ILD.

**Methods:**

A total of 239 RA patients fulfilling the American Rheumatism Association (ACR) 1987 revised criteria were enrolled and subclassified as RA-ILD and RA-nILD based on the results of high-resolution computed tomography scans (HRCT) of the chest. The disease activity of RA was assessed by Disease Activity Score for 28 joints (DAS28) and categorized as high, moderate, low, and remission. Chemiluminescence immunoassays were applied to determine the serum levels of CHI3L1. Univariate analysis was performed and the receiver operating characteristics (ROC) curves were plotted to evaluate the correlation between RA-ILD and CHI3L1.

**Results:**

Among the eligible RA patients studied, 60 (25.1%) patients were diagnosed with RA-ILD. Compared with RA-nILD, RA patients with ILD had significantly higher median age (median [IQR], 68.00 [62.00-71.75] vs 53.00 [40.00-63.00], p<0.001) and a higher proportion of males (21 (35.0%) vs 30 (16.8%), p=0.003). Notably, differences in DAS28 scores between the two groups were not observed. The serum level of CHI3L1 was significantly higher in RA-ILD patients (median [IQR], 69.69 [44.51-128.66] ng/ml vs 32.19 [21.63-56.99] ng/ml, p<0.001). Furthermore, the areas under the curve (AUC) of CHI3L1 attained 0.74 (95% confidence interval [CI], 0.68-0.81, p<0.001) in terms of identifying patients with RA-ILD from those without ILD. Similar trends were seen across the spectrum of disease activity based on DAS28-ESR.

**Conclusion:**

Our findings of elevated serum CHI3L1 levels in RA-ILD patients suggest its possible role as a biomarker to detect RA-ILD noninvasively.

## Introduction

Rheumatoid arthritis (RA) is a prevalent chronic rheumatic disease that can cause progressive articular damage and extra-articular manifestations, including pulmonary involvement, vasculitis, rheumatoid nodules, and systemic comorbidities (1). Interstitial lung disease (ILD) is the most common pulmonary manifestation, occurring in 10% of patients with RA (2). ILD is strongly associated with poor prognosis and is the common cause of RA mortality, secondary only to cardiovascular diseases (3, 4). A recent study shows that the 1- and 5-year mortality rates of rheumatoid arthritis-associated ILD (RA-ILD) were 13.9% and 39.0%, while these rates were 3.8% and 18.2% in RA patients without ILD (RA-nILD) (5). Moreover, despite decreasing mortality rates of RA, there has been an increase in deaths from RA-ILD (2), thus prompting a great effort to achieve an earlier diagnosis and improve the prognosis.

High-resolution chest computed tomography (HRCT) is accepted as the gold-standard approach for the diagnosis of ILD (6). Although showing abnormalities in 19% of patients with RA (6), HRCT of the lungs is not performed for routine RA diagnoses in consideration of radiation exposure and high cost (7), which is not conducive to early detection of RA-ILD. Early recognition and monitoring are crucial to provide better treatment and improve the prognosis. Given the impressive advances in the development of molecular detection techniques, the discovery and validation of disease-specific biomarkers could be of great clinical importance (8).

Antibodies and lung epithelium-specific biomarkers play a crucial role in predicting RA-ILD (9). Rheumatoid factor (RF) and anti-citrullinated peptide antibodies (ACPA), two serologic biomarkers for RA included in the European League Against Rheumatism (EULAR)/American College of Rheumatology (ACR) 2010 criteria (10), have been demonstrated to act as biomarkers of RA-ILD (11, 12). Other antibodies have also been proven to have strong associations with RA-ILD, such as anti-malondialdehyde-acetaldehyde (anti-MAA) (13) and anti-carbamylated protein (anti-CarP) (14). In addition, inflammatory mechanisms may cause repetitive injuries to alveolar epithelial cells (AECs), such as the apoptosis of ACE type I (ACE I) and the hyperplastic of AEC type II (ACE II). The regenerating ACE II could produce and recruit a series of cytokines and growth factors, and release surfactant proteins (15), which have potential predictive value. Factors like Krebs von den Lungen-6 (KL-6) (16), matrix metalloproteinase (MMP)-7 (17), and C-X-C motif chemokine 10 (CXCL10) (17) have been demonstrated to aid RA-ILD identification.

Chitinase-3 like-protein-1 (CHI3L1) plays a vital role in tissue repair, inflammation, and remodeling responses. It is synthesized and secreted by a multitude of cells including macrophages, neutrophils, synoviocytes, chondrocytes, fibroblast-like cells, and tumor cells (18–21). CHI3L1 has been regarded as a promising biomarker for predicting and evaluating the severity of RA (21, 22). In addition, serum CHI3L1 levels are significantly higher in ILD patients (23). It plays a profibrotic role in lung fibrosis repair phase by augmenting alternative macrophage activation, fibroblast proliferation, and matrix deposition (24). However, the associations between CHI3L1 and RA-ILD remain unknown.

The purpose of this study is to evaluate whether CHI3L1 can identify the presence of RA-ILD in RA patients. Additionally, we will assess its diagnostic capacity for ILD in patients with different disease activities of RA.

## Methods

### Study design and participants

We performed a large, single-center, retrospective observational study of 239 RA patients who received serum CHI3L1 test in the Department of Rheumatology of Peking Union Medical College Hospital (Beijing, China) from 2019 to 2021. Diagnosis of RA was based on the American Rheumatism Association (ACR) 1987 revised criteria (25) and ILD was evaluated through chest CT scans. Patients with other autoimmune diseases, other chronic lung diseases, liver damage, cancers, infections, or had a history of ILD before the diagnosis of RA were excluded. Patients were divided into two subgroups of RA-ILD (60, 25.1%) or RA-nILD (179, 74.9%) according to the complication of ILD. Articular disease activity was quantified and categorized by the disease activity score in 28 joints (DAS28) assessment (26). Baseline demographics were acquired from the medical records. This study was approved by the Medical Ethics Committee of Peking Union Medical College Hospital (PUMCH) (ethics number I-22PJ457). All participants provided written informed consent.

### RA-ILD diagnosis

RA-ILD was diagnosed according to the presence of typical features in the lungs using HRCT. The features included irregular linear or reticular opacities, ground-glass opacities, consolidation, honeycombing, septal thickening, and traction bronchiectasis or bronchiolectasis according to the consensus for idiopathic interstitial pneumonia of the American Thoracic Society/European Respiratory Society (ATS/ERS) (27). Diagnoses were confirmed by expert radiologists in a blinded manner.

### Scoring

Two disease activity score (DAS) standards, DAS28-ESR (erythrocyte sedimentation rate) and DAS28-CRP (C reactive protein), were applied to categorize disease activity states of RA patients as high (DAS28>5.1), moderate (3.2<DAS28 ≤ 5.1), low (2.6<DAS28 ≤ 3.2) disease activity and remission (DAS28 ≤ 2.6) (26).

### Chemiluminescence immunoassay

Serum levels of CHI3L1 were analyzed by a commercial test system, iFlash CLIA kits (YHLO Biotech Co., Shenzhen, China, Y-CLIA for short) that had been proved a suitable choice for the Chinese population in our previous studies (28). More specifically, Y-CLIA conducted paramagnetic particle chemiluminescent immunoassay using a fully automated iFlash 3000 Chemiluminescence Immunoassay Analyzer. Serum samples were obtained by separation from peripheral blood and stored at −80°C until use. No sample was exposed to more than one freeze-thaw cycle before analysis.

### Statistical analyses

For the comparison of age, sex, DAS28-ESR score, DAS28-CRP score, and serum CHI3L1 levels between RA-ILD and RA-nILD, we used Chi-squared test and Mann-Whitney U test where appropriate. Kruskal-Wallis tests were used to compare the serum CHI3L1 levels within the groups categorized according to the disease activity of RA. Receiver operating characteristic (ROC) curves and areas under the curve (AUC) were generated to determine the efficacy of serum CHI3L1 levels in distinguishing individuals with RA-ILD from RA-nILD and determining the optimum cut-off level. All analyses were performed using SPSS, version 26.0. P value less than 0.050 was considered statistically significant.

## Results

The demographic, clinical, and laboratory features of the 239 RA patients are summarized in **Table 1**. Among all enrolled RA patients, 60 (25.1%) showed definite ILD features on chest HRCT. Compared with patients without ILD features, those with ILD were more likely to be older (median [IQR], 68.00 [62.00-71.75] vs 48.00 [37.00-57.00], p<0.001), have a higher proportion of males (21 (35.0%) vs 30 (16.8%), p=0.003), and have high titer positivity of RF or/and anti-CCP (55 [91.7%] vs 137 [76.5%]). Furthermore, the age of RA patients and CHI3L1 levels were moderately correlated, and the relationship was statistically significant revealed by *Pearson Correlation Coefficient* (r(237) = 0.323, p < 0.01). Different age groups were divided and patients older than 65 were more likely to have elevated CHI3L1 than 50-55 years old patients, as same as 60-65 and 30-40 years old patients (**Supplementary Figure 1**). However, there was no statistical difference between gender and CHI3L1 levels (**Supplementary Figure 2**). The presence of RA-ILD and DAS28 scores might have no relationship, neither DAS28-ESR (p=0.732) nor DAS28-CRP (p=0.202). In addition, the disease duration was similar in RA patients with and without ILD (median [IQR], 5.56 [1.06-18.31] vs 5.10 [1.68-10.16], p=0.548). Among the baseline therapies, patients with ILD were more likely to be treated with glucocorticoids (9 (90%) vs 85 (47.5%), p=0.010).

**Table 1:** Baseline characteristics of the study population.

Characteristics	All patients	RA-ILD	RA-nILD	p-value
Patients, n (%)	239 (100)	60 (25.1)	179 (74.9)	/
Age, median (IQR), years	53.00 (40.00-63.00)	68.00 (62.00-71.75)	48.00 (37.00-57.00)	<0.001
Female, n (%)	188 (78.7)	39 (65.0)	149 (83.2)	0.003
DAS28-ESR, median (IQR)	3.370 (2.240-5.170)	3.180 (2.340-5.190)	3.540 (2.198-5.170)	0.732
DAS28-CRP, median (IQR)	2.840 (1.855-4.545)	2.430 (1.710-4.210)	3.030 (1.950-4.710)	0.202
High titer positivity of RF or/and anti-CCP*, n(%)	192 (80.3)	55 (91.7)	137 (76.5)	0.009
Low titer positivity of RF or/and anti-CCP*, n(%)	52 (21.8)	9 (15.0)	43 (24.0)	0.153
CHI3L1, median (IQR), ng/mL	40.68 (23.71-71.72)	69.69 (44.51-128.7)	32.19 (21.63-56.99)	<0.001
No. of swollen joints*, median (IQR)	2.00 (0.00-5.25)	2.00 (0.50-8.00)	2.00 (0.00-5.50)	0.800
Duration of RA*, median (IQR), years	5.10 (1.55-10.22)	5.56 (1.06-18.31)	5.10 (1.68-10.16)	0.548
DAS28-ESR				
High (n=60)	49.37 (30.55-72.45)	59.54 (46.32-96.49)	42.09 (24.31-62.72)	0.034
Moderate (n=61)	39.87 (23.23-67.19)	110.5 (48.09-172.9)	31.95 (22.27-55.93)	<0.001
Low (n=26)	49.26 (25.96-77.70)	76.09 (49.19-135.1)	29.47 (24.75-60.55)	0.029
Remission (n=81)	32.75 (21.07-66.84)	61.30 (28.08-93.30)	30.21 (18.21-52.17)	0.004
DAS28-CRP				
High (n=39)	49.10 (27.93-66.20)	89.71 (55.31-129.5)	48.13 (28.55-62.85)	0.147
Moderate (n=67)	41.74 (22.73-67.78)	59.31 (44.47-138.0)	31.95 (21.88-56.77)	0.001
Low (n=27)	32.24 (22.15-65.67)	189.2 (5.470-353.6)	31.16 (22.85-61.40)	0.220
Remission (n=97)	37.19 (23.23-74.14)	53.33 (26.20-95.82)	30.60 (18.01-54.35)	0.026
Treatment*, n (%)				
Glucocorticoids	94 (49.7)	9 (90.0)	85 (47.5)	0.010
Methotrexate	132 (69.8)	6 (60.0)	126 (70.4)	0.492
Leflunomide	47 (24.9)	4 (40.0)	43 (24.0)	0.269
TNFα inhibitor	40 (21.2)	1 (20.0)	39 (21.8)	0.691

The categorical variables were compared by Chi-squared test, and Mann-Whitney U test was used for non-normally distributed continuous variables. Kruskal-Wallis tests were used to compare within groups.

RA-ILD, rheumatoid arthritis-associated interstitial lung disease; RA-ILD, rheumatoid arthritis without interstitial lung disease; IQR, interquartile range; SD, standard deviation; DAS28, disease activity score 28 joints; ESR, erythrocyte sedimentation rate; CRP, C reactive protein; CHI3L1, Chitinase-3 like-protein-1.

*189 patients (10 RA-ILD, 179 RA-nILD) were included.

Data missing: RA-ILD: DAS28-ESR (n=1), DAS28-CRP (n=1). RA-nILD: DAS28-ESR (n=10), DAS28-CRP (n=9).

The symbol "/" indicates that the value is empty.

### Relationship between the presence of RA-ILD and serum levels of CHI3L1

**Figure 1:**
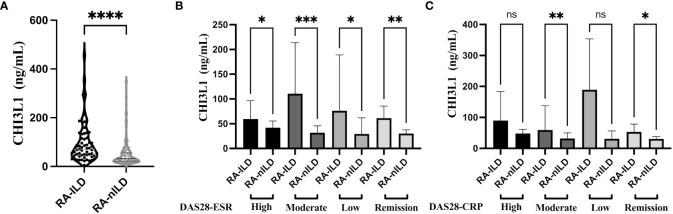
Serum level of CHI3L1 between RA-ILD and RA-nILD and among different disease activity subgroups. **(A)** RA patients with or without ILD. **(B)** RA patients with different activity states according to DAS28-ESR. **(C)** RA patients with different activity states according to DAS28-CRP. Violin plot depicts levels of CHI3L1 in individual serum samples from RA-ILD and RA-nILD. The dotted lines represent median and quartiles. The error bars show the median with 95% CI. *P* values were determined by Mann-Whitney U test **(A)** and Kruskal-Wallis test **(B, C)**. *P < 0.05, **P < 0.01, ***P < 0.001, ****P < 0.0001. "ns" means "no significance".

The CHI3L1 levels were significantly higher in the RA-ILD than in the RA-nILD group (**Table 1**; **Figure 1A**). Compared with RA-nILD (median [IQR], 32.19 [21.63-56.99] ng/mL), the serum CHI3L1 levels were increased in RA-ILD patients (median [IQR], 69.69 [44.51-128.66] ng/ml, p<0.001). In addition, statistically significant elevations of CHI3L1 were observed across the spectrum of disease activity based on DAS28-ESR, and in moderate and low disease activity groups based on DAS28-CRP (**Table 1**; **Figures 1B, C**).

### Efficiency of serum CHI3L1 levels as biomarkers for RA-ILD

**Figure 2:**
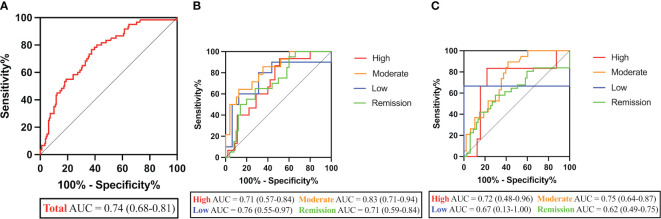
Performance characteristics of CHI3L1 levels in RA-ILD diagnosis. **(A)** ROC curves of CHI3L1 levels in RA-ILD and RA-nILD patients. **(B)** ROC curves of CHI3L1 levels in groups at different disease activity states based on DAS28-ESR. **(C)** ROC curves of CHI3L1 levels in groups at different disease activity states based on DAS28-CRP. AUC and 95% CI are shown in parentheses.

Further demonstrating the predictive potential of CHI3L1 as a biomarker for RA-ILD, ROC assessment revealed strong performance characteristics with AUC of 0.74 (95% CI, 0.68-0.81, p<0.001) (**Figure 2A**). The cross-validation of the ROC curve was performed for further confirmation (**Supplementary Figure 3**). A positive cut-off value was defined as ≥44.20 ng/mL according to the sensitivity (76.67%), specificity (62.57%), and Youden Index. CHI3L1 positivities were more frequent in RA-ILD patients (46 [76.67%] vs 70 [39.11%], p=0.006). Based on logistic regression analysis, however, only older age was found to be significantly associated with RA-ILD (p<0.001), while CHI3L1 levels (p=0.415) and male sex (p=0.074) were not.

As shown in **Figure 2B**, the AUCs for CHI3L1 in the high/moderate/low/remission RA disease activity group based on DAS28-ESR were 0.71 (p=0.018), 0.83 (p<0.001), 0.76 (p=0.031), and 0.71 (p=0.006), respectively. However, there was no significant difference between the curves in high (p=0.093), low (p=0.355), and remission (p=0.062) disease activity groups based on DAS28-CRP (**Figure 2C**; **Table 2**).

**Table 2:** ROC Curves of CHI3L1 levels for RA-ILD patients at different RA activity.

	AUC	95% CI	Diagnostic threshold (ng/mL)	No. of CHI3L1 seropositive	Sensitivity (%)	Specificity (%)	P value
DAS28-ESR							
High	0.71	0.5656 - 0.8448	40.04	38	93.33	48.89	0.018
Moderate	0.83	0.7071 - 0.9447	67.39	16	64.29	87.50	<0.001
Low	0.76	0.5457 - 0.9668	49.26	14	80.00	68.75	0.031
Remission	0.71	0.5858 - 0.8382	43.67	28	65.00	72.00	0.006
DAS28-CRP							
High	0.72	0.4789 - 0.9586	64.60	13	83.33	78.13	0.093
Moderate	0.75	0.6376 - 0.8701	37.60	38	89.47	58.33	0.001
Low	0.67	0.1332 - 1.000	119.1	3	66.67	100.00	0.355
Remission	0.62	0.4877 - 0.7477	55.64	33	58.06	70.15	0.062

AUC, area under the curve; CI, confidence interval; DAS28, disease activity score 28 joints; ESR, erythrocyte sedimentation rate; CRP, C reactive protein.

## Discussion

To our knowledge, this is the first study to analyze the association between CHI3L1 and RA-ILD. We found that higher levels of serum CHI3L1 were measured among patients with radiographic evidence of ILD features compared with RA patients without lung involvement. Additionally, older age and male sex were associated with RA-ILD. In particular, CHI3L1 exhibited the valuable diagnostic ability to identify the presence of ILD in patients with different disease activities of RA.

Survival of RA has been improved in the last few decades, along with the changes in treatment paradigms, early intervention, and treat-to-target approaches (29). The declined mortality rates are mostly due to a decrease in cardiovascular mortality while deaths from ILD remain significant (30). Considering that respiratory symptoms are not always apparent in RA-ILD (31), early detection and management are crucial to improving the prognosis and survival. The relationship between CHI3L1 and RA or ILD has been addressed in different studies (21, 22, 24, 32), while no association between CHI3L1 and RA-ILD has been confirmed.

Consistent with multiple studies (33, 34), the association of RA-ILD with older age and male sex is demonstrated in this study. Older age is associated with RA-ILD suggesting that immunosenescence and telomere shortening may be linked with RA-ILD risk (35). Notably, it seems somewhat paradoxical that RA is more prevalent in females than males, with a female-to-male sex ratio ranging from 4:1 to less than 2:1 in younger and older patients respectively (36). The distinctions might be explained partly by different exposure to environmental risk factors like smoking (37), which is involved in chronic mucosal inflammation of lung and plays a positive role in the development of RA-ILD (38). Some studies have demonstrated that smoking might induce citrullination of peptide antigens in the lungs via peptidyl arginine deiminase-2 (PAD2) (39, 40). Moreover, increased PAD2 was observed in the broncho-alveolar lavage (BAL) fluid of smokers compared to non-smokers (41). In addition, higher level of ACPA has been found among RA patients with ILD (12). It seemed that smoking might partly explain the higher proportion of male RA-ILD patients. Furthermore, several studies suggest that gonadal hormones (36), genetic background (42–44), and other confounding factors (44) also contribute to RA-ILD. The role of aging and gender in RA-ILD remains to be clarified. Traditional disease-modifying antirheumatic drugs (DMARDs) like methotrexate are the basis of therapeutic intervention in most patients with RA, while the effects on the presence of RA-ILD remain controversial (45). Treating with methotrexate seemed not to affect the arise of ILD but glucocorticoids did in this study.

The disease activity of RA determined by DAS28 appears to have no influence on the presence of RA-ILD, indicating the lack of association between the disease activity of RA and RA-ILD. In contrast, a cross-sectional study identified mean DAS28 as an independent predictor for preclinical RA-ILD (odds ratio (OR)=2.0, 95% CI [1.2-3.4]) (46). However, the sample size of this study is smaller than ours, which might contribute to the variance. A previous prospective cohort study conducted in the UK with 1460 RA patients involved shared similar results with ours that the DAS28 score at baseline had no association with RA-ILD (hazard ratio (HR)=1.11, 95% CI [0.94-1.31]) (47). The difference among these studies may come from differences in patient population, exclusions of confounding factors, and disease definition.

Mann-Whitney U test and AUC analysis showed a robust relationship between CHI3L1 level and the presence of RA-ILD, suggesting the potential role of CHI3L1 as a biomarker and its immune response on RA-ILD development. So far, no studies have evaluated CHI3L1 levels in RA-ILD patients while CHI3L1 have been detected in RA (22) patients and ILD patients (32). Several serum biomarkers have been proposed for RA-ILD diagnosis, including antibodies (ie. RF and ACPA) (11, 12) and a range of cytokines and growth factors released by active specialized macrophages and stromal cells (ie. MMP-7, surface protein D, and KL-6) (16, 17). Given the similarities in clinical, radiographic, and genetic characteristics shared by RA-ILD and idiopathic pulmonary fibrosis (IPF), several studies have noted that the biomarkers might repurpose from one to the other (48). For instance, serum MMP-7 and ACPA levels which are significantly higher in IPF than controls are also elevated in RA-ILD compared to those with RA without ILD (17, 49–51). Interestingly, the serum levels of CHI3L1 could distinguish between IPF and controls (245.8 ± 180.2 ng/mL vs 116.0 ± 58.3 ng/mL, p<0.001) and predict survival (52). Considering the biological activities of CHI3L1 determined in RA and hepatic fibrosis such as stimulating the growth of fibroblasts (19), driving macrophage activation and differentiation (53), and regulating the ECM (54), it may have a significant impact on the potential pathobiology of both IPF and RA-ILD. In addition, the data from our study suggested that CHI3L1 was valuable for the diagnosis of RA-ILD in all spectrums of disease activity based on the DAS28-ESR score, and in moderate and low disease activity groups based on DAS28-CRP. It seems that applying DAS28-ESR as the scoring standard may increase the accuracy of RA-ILD assessment.

Our study has some limitations. First, patients’ smoking history and specific autoantibodies concentration are not included; thus, these confounders may introduce bias to our results. Cigarette smoking could increase the risk of RA-ILD with a dose effect relationship (55). Second, this is a single-center study focused on RA patients, which limits the representativeness of the results. In the future, large sample and multi-center studies are needed to evaluate the link between CHI3L1 and RA-ILD. Third, several studies have shown that the efficiency of biomarkers varies in different disease statuses of RA-ILD (17, 56), while our study did not assess the severity of ILD. Moreover, the inclusion of RF, ACPA, and other potential biomarkers determined by previous studies tends to improve the accuracy of the prediction model (16, 17, 56). Additionally, comparing and contrasting the diagnostic ability of CHI3L1 with validated indicators such as KL-6 and HRCT scores might help verify its potency as a biomarker. Finally, there were no hold-out test sets or external validation cohorts.

In conclusion, an association between CHI3L1 and RA-ILD was found. It poses the debate of whether CHI3L1 could contribute to the development of this devastating extra-articular manifestation. Future studies will help define the potential underlying pathological mechanism of this connection.

## Data availability statement

The raw data supporting the conclusions of this article will be made available by the authors, without undue reservation.

## Ethics statement

The studies involving humans were approved by Medical Ethics Committee of Peking Union Medical College Hospital (ethics number I-22PJ457). The studies were conducted in accordance with the local legislation and institutional requirements. Written informed consent for participation in this study was provided by the participants’ legal guardians/next of kin.

## Author contributions

All authors were involved in the conception and design of this study. RYu, XL, XD, SL, and YW contributed to the performance of experiments. QW and XT assisted in the collection and preservation of blood samples. YZ, DK, and RYan collected and evaluated patient data. QW, XT, and ML assisted in the recruitment of patients. RYu and XL analyzed the data and wrote the first draft of the manuscript. XD, SL, and YW contributed to validation. YZ, DK, RYan, and ML assisted in supervision of general work. All authors contributed to manuscript revision, read, and approved the submitted version.

## Supplementary material

Supplementary Figure 1 Serum level of CHI3L1 among different age groups. *P* values were determined by the Kruskal-Wallis test. ***P* < 0.01

Supplementary Figure 2 Serum level of CHI3L1 between male and female patients. *P* values were determined by the Independent t-test. The dotted lines represent median and quartiles.

Supplementary Figure 3 Cross-validation of ROC curves assessing the ability of CHI3L1 levels in RA-ILD diagnosis. The AUC of 100% training was 0.735, of 70% training was 0.692, and of K-Fold was 0.749.
