# Supplementary figures and images for: Serum CHI3L1 as a biomarker of interstitial lung disease in rheumatoid arthritis
Source: Front Immunol. 2023 Aug 17;14:1211790. doi: 10.3389/fimmu.2023.1211790 (PMC10469784; doi:10.3389/fimmu.2023.1211790)

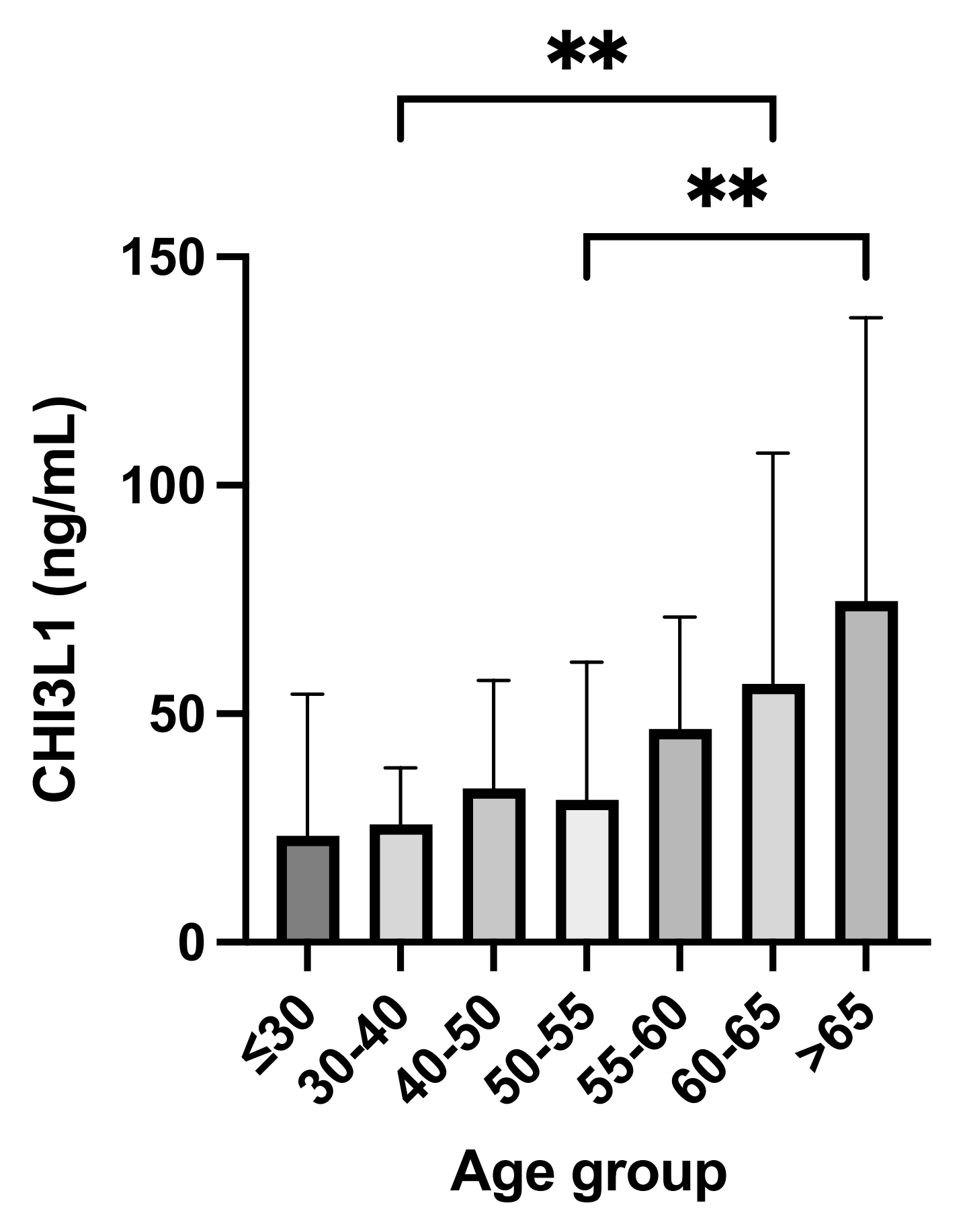

Supplement: Supplementary Figure 1 — Serum level of CHI3L1 among different age groups. P values were determined by the Kruskal-Wallis test. **P < 0.01 [file Image_1.tif]

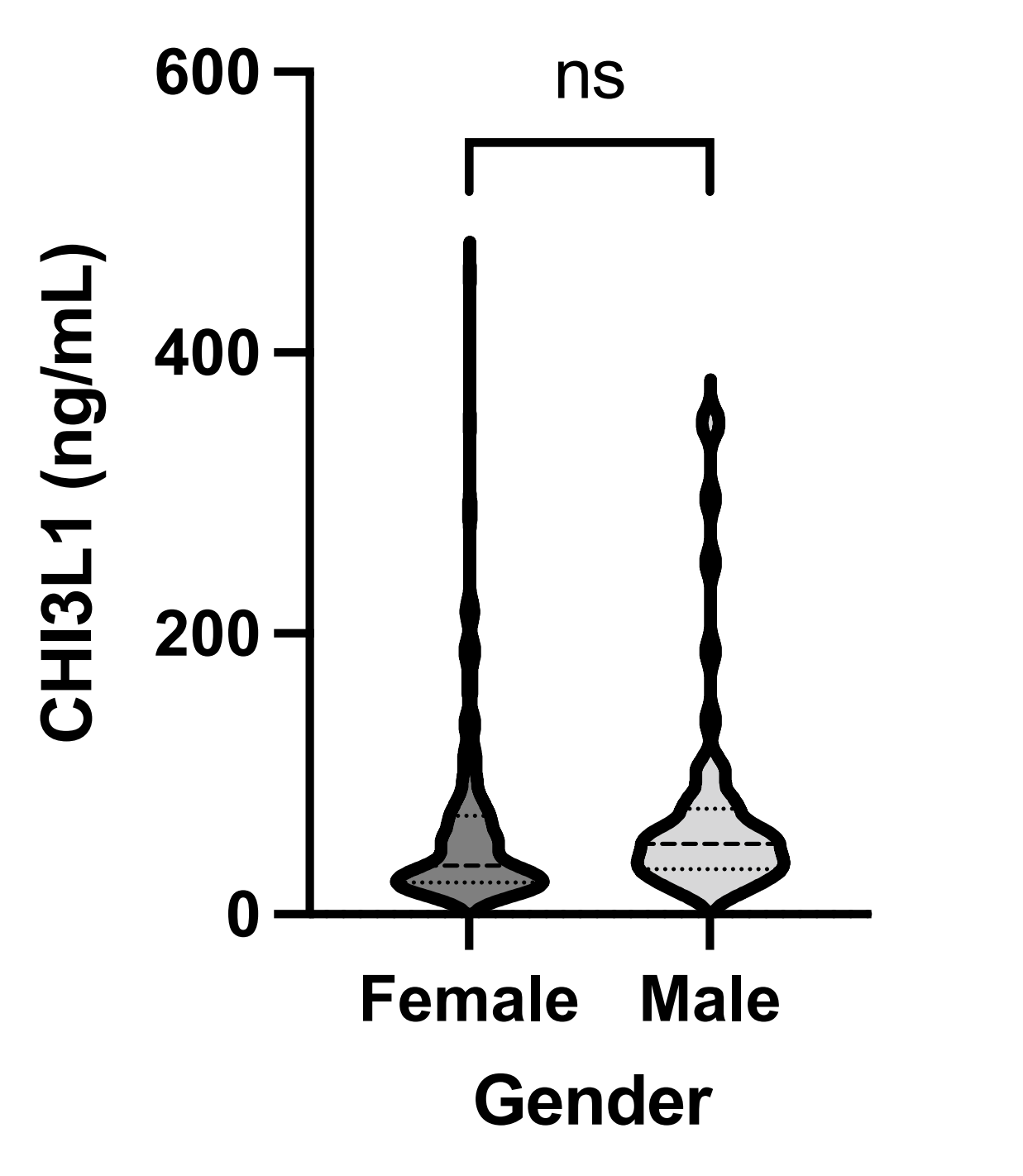

Supplement: Supplementary Figure 2 — Serum level of CHI3L1 between male and female patients. P values were determined by the Independent t-test. The dotted lines represent median and quartiles. [file Image_2.tif]

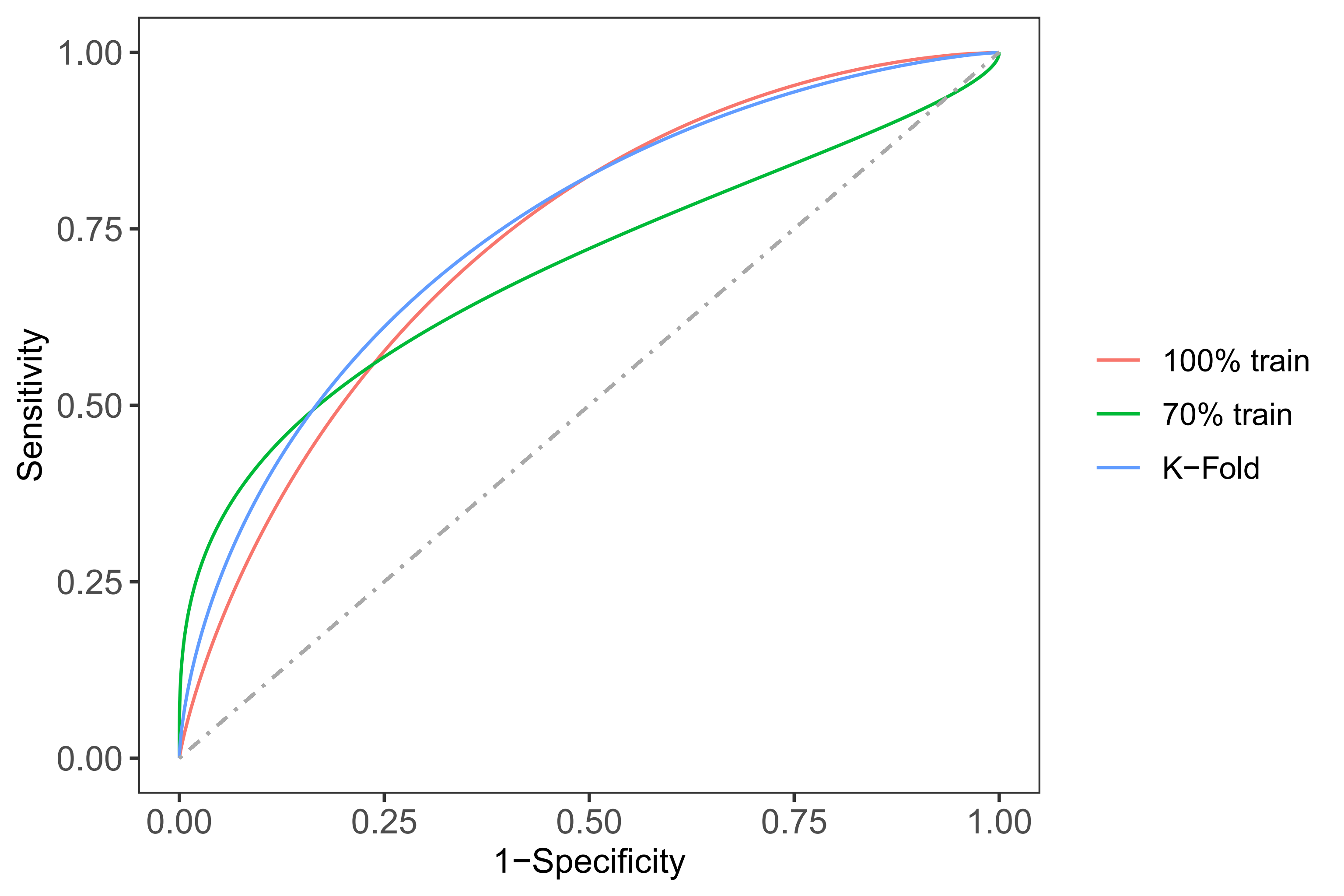

Supplement: Supplementary Figure 3 — Cross-validation of ROC curves assessing the ability of CHI3L1 levels in RA-ILD diagnosis. The AUC of 100% training was 0.735, of 70% training was 0.692, and of K-Fold was 0.749. [file Image_3.tif]
